# The Potential Role of Apigenin in Cancer Prevention and Treatment

**DOI:** 10.3390/molecules27186051

**Published:** 2022-09-16

**Authors:** Arshad Husain Rahmani, Mohammed A. Alsahli, Ahmad Almatroudi, Mashael Abdullah Almogbel, Amjad Ali Khan, Shehwaz Anwar, Saleh A. Almatroodi

**Affiliations:** 1 Department of Medical Laboratories, College of Applied Medical Sciences, Qassim University, Buraydah 52571, Saudi Arabia; 2 Department of Basic Health Science, College of Applied Medical Sciences, Qassim University, Buraydah 52571, Saudi Arabia

**Keywords:** apigenin, cancer, cell signalling pathways, bioavailability, synergistic effects

## Abstract

Cancer is the leading cause of death worldwide. In spite of advances in the treatment of cancer, currently used treatment modules including chemotherapy, hormone therapy, radiation therapy and targeted therapy causes adverse effects and kills the normal cells. Therefore, the goal of more effective and less side effects-based cancer treatment approaches is still at the primary position of present research. Medicinal plants or their bioactive ingredients act as dynamic sources of drugs due to their having less side effects and also shows the role in reduction of resistance against cancer therapy. Apigenin is an edible plant-derived flavonoid that has received significant scientific consideration for its health-promoting potential through modulation of inflammation, oxidative stress and various other biological activities. Moreover, the anti-cancer potential of apigenin is confirmed through its ability to modulate various cell signalling pathways, including tumor suppressor genes, angiogenesis, apoptosis, cell cycle, inflammation, apoptosis, PI3K/AKT, NF-κB, MAPK/ERK and STAT3 pathways. The current review mainly emphases the potential role of apigenin in different types of cancer through the modulation of various cell signaling pathways. Further studies based on clinical trials are needed to explore the role of apigenin in cancer management and explain the possible potential mechanisms of action in this vista.

## 1. Introduction

**Figure 1:**
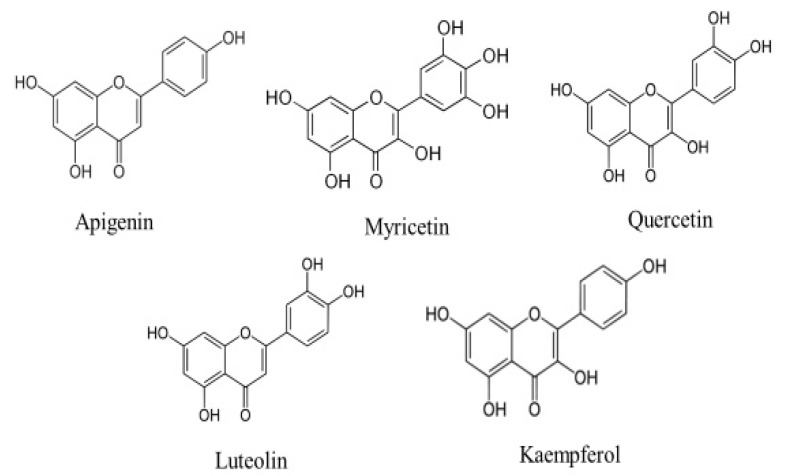
Chemical structures of flavonoids, such as apigenin, myricetin quercetin, luteolin and kaempferol.

Cancer is the leading cause of death worldwide. It is accountable for around 7.6 million deaths worldwide, which is predicted to increase to 13.1 million by 2030 [1]. In spite of advances in the treatment of cancer, currently used treatment modules remain ineffective, cause’s adverse effect and effective cancer therapy still needs to be established. These days, health sciences researchers have their primary attention on medicinal plants or natural compounds with anti-cancer properties to improve the cancer problems. Accumulating evidence from previous studies designate that dietary components may significantly alter the natural history of carcinogenesis, and that a reverse correlation between a high consumption of fruits as well as vegetables and the incidence of some cancers does exist [2,3]. Amongst the herbs, flavonoids are a huge subgroup of the family of natural polyphenolic compounds that are the consequence of secondary metabolism in plants [4]. Currently, scientists have proven that flavonoids have been effective in the prevention as well as the control of common disease complexes, including cancer [5]. Flavonoids have been considered to have scientific interest, as they show numerous pharmalogical activities such as anti-oxidant activity, anti-inflammation, free radical scavenging, and anti-cancer activity, regulating cellular proliferation, induction of apoptosis, preventing platelet aggregation as well as reducing plasma levels of low-density lipoproteins [6,7,8,9]. Among the over 6000 different flavonoids, apigenin, myricetin quercetin, luteolin and kaempferol are the five most ubiquitous plant flavonoids [10] (Figure 1). Apigenin (APG) is a consumable flavonoid (4′,5,7-trihydroxyflavone) that has been become popularized as a health-promoting drug in recent years owing to its poor intrinsic toxicity and distinct activities on normal versus cancer, compared with other structurally related flavonoids [11]. The powerful anti-oxidant as well as anti-inflammatory activities of apigenin are a considerable reason for its probable cancer preventive effects [12]. More significantly, apigenin meaningfully participates in the prevention of cancer through the induction of apoptosis in various cell lines as well as animal models [13]. In this review, we have outlined the substantial therapeutic implications of apigenin in various types of cancer. This study also looked at its mechanisms of action, with a focus on cell signalling pathways.

## 2. Major Mechanisms of Apigenin Involved in Cancer Management

**Figure 2:**
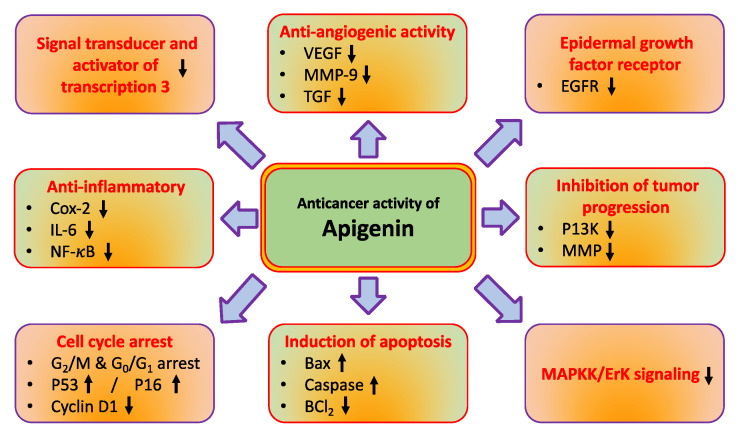
Apigenin’s role in cancer management through modulating cell signaling pathways. The complex cell nature of cancer is characterised by a number of complex molecular interactions and mechanisms.

The role of natural compounds in cancer prevention has been through modulating cell signalling pathways [14,15]. Apigenin regulates different molecular pathways such as tumor suppressor genes, angiogenesis, and nuclear factor-κB, and induces autophagy, cell cycle and, apoptosis (Figure 2 and Table 1). The major mechanisms of apigenin in the inhibition of cancer are described below.

### 2.1. Inflammation

Inflammation is one of the foremost features to endorse the progression of tumor, increases cancer risk [35]. Furthermore, during the tumor expansion, inflammatory mediators together with reactive nitrogen species, reactive oxygen species, and cytokines derived from tumor-infiltrating immune cells causes the initiation of epigenetic changes in silent tumor suppressor genes as well as pre-malignant ones [36]. Flavonoids including apigenin and epigallocathechin gallate have been revealed to inhibit the activation of immune cells and the downstream chemokines and cytokines [37], thus it may measure as a natural inhibitor and can stop the activation of an innate and adaptive immune system [38]. 

TNFα stimulus leads to the huge increase of chemotactic protein CCL2, granulocyte macrophage colony-stimulating factor (GMCSF), and IL-1α and IL-6, all blocked by the treatment of apigenin. Furthermore, the data demonstrate that the decrease of CCL2 via apigenin treatment in the presence TNFα paralleled the decrease of phosphorylated extracellular signal-regulated kinase 1. [16]. TNFα stimulation leads to a large rise in CCL2, granulocyte macrophage colony-stimulating factor (GMCSF), IL-1α and IL-6, all suppressed by apigenin [16]. Apigenin can regulate the production as well as the gene expression of mucin via regulating NF-κB signaling pathways in airway epithelial cells [17].

### 2.2. Tumor Suppressor Gene

Apigenin effectiveness on primary effusion lymphoma (PEL) has been investigated. It was reported that that apigenin induced primary effusion lymphoma cell death and autophagy in addition to a substantial decrease in intracellular reactive oxygen species. Mechanically, apigenin has been found to activate p53 that is reported to upregulate catalase, and to inhibit STAT3, the most significant pro-survival pathway in PEL, as assessed by p53 silencing [31]. A549 cells were employed in a study to see if apigenin treatment resulted in the accumulation of p53. Increased p53 protein accumulation has been reported in samples that were treated with apigenin. Furthermore, apigenin has been reported to induce phosphorylation of p53 in a dose-dependent manner. In addition, the combination of apigenin and cisplatin has been shown to dramatically increase the levels of p53 protein as compared to cisplatin alone. 

However, the concentrations of p53 mRNA in A549 cells administered with apigenin and cisplatin did not change significantly, suggesting that increased p53 protein expression was probably linked with reduced degradation. Apigenin magnified the cytotoxic impact of cisplatin by inducing p53 accumulation and p53-regulated proapoptotic gene expression, suggesting that apigenin amplified the cytotoxic effect of cisplatin via the induction of p53 accumulation and p53-regulated proapoptotic gene expression [18]. 

### 2.3. Induction of Apoptosis

Apigenin plays a significant role in the induction of apoptosis. Apigenin administration resulted in a substantial decrease in cell viability as well as the activation of apoptosis with a time-dependent. Apigenin actions were further exacerbated by a reduction in Bcl-xL and Bcl-2, as well as an elevation in the activated state of the Bax protein [19]. Apigenin induced apoptosis via the activation of the mitogen-activated protein kinase (MAPK) signaling pathway, as well as downregulated Srx expression in cutaneous squamous cell carcinoma cell lines [39]. Moreover, another study result reported that apigenin also potentiated 5-fluorouracil-induced apoptosis of HCT116 cells as well as enhanced cell cycle disruption. Additionally, apigenin increased intracellular and intramitochondrial Ca^2+^ concentrations, reactive oxygen species production, and mitochondrial membrane potential upon cotreatment with 5-fluorouracil. Knockdown of forkhead box protein M, a transcription factor controlling 5-fluorouracil sensitivity, enhanced the potentiation of apoptosis by apigenin in cancer cells [40]. The efficiency of a combination of apigenin and Bcl-2 inhibitor HA14-1 (HA) was tested in human malignant neuroblastoma cells. Dose-response studies indicated that treatment with HA and apigenin synergistically reduced cell viability in human malignant neuroblastoma. Moreover, combination therapy downregulated angiogenic factors and also induced the extrinsic pathway of apoptosis with the activation of caspase-8 for Bid cleavage to tBid [41]. Flavonoids were found to enhance the proteolytic cleavage of poly-(ADP-ribose) polymerase and to induce a rapid elevation of caspase-3 activity (PARP). These flavonoids also caused a decrease in mitochondrial transmembrane potential, an increase in reactive oxygen species formation, the discharge of mitochondrial cytochrome c into the cytosol, and the processing of procaspase-9. Furthermore, procaspase-9 processing, the cytochrome c release to the cytosol, and a caspase-3-dependent pathway, all contribute to flavonoid-induced apoptosis [42]. PARP cleavage DNA fragmentation revealed that apoptosis was induced by apigenin treatment. These effects were linked to a change in the Bax/Bcl-2 ratio in favor of apoptosis [20].

### 2.4. Angiogenesis

Apigenin inhibited lung cancer cell proliferation and vascular endothelial growth factor (VEGF) transcriptional activation in a dose-dependent way. The mechanism involved in this process is explained as apigenin-inhibited VEGF transcriptional activation through the hypoxia-inducible factor 1 binding site and specifically decreased HIF-1alpha. In vivo based findings reported that apigenin significantly inhibited tumor growth in nude mice. Moreover, apigenin inhibited HIF-1alpha and VEGF expression in the tumor tissues, showing an inhibitory effect of apigenin on angiogenesis [21]. Apigenin showed a role in the inhibition of the hypoxia-induced expression of vascular endothelial growth factor mRNA in human umbilical artery endothelial cells. Moreover, the effect of apigenin on the dealings of HIF-1α with heat shock protein 90 was examined, which is described to be important for the maintenance of HIF-1α, and established that the expression of vascular endothelial growth factor was inhibited through the degradation of HIF-1α via interfering with the Hsp90 function [22]. The effects and mechanism of apigenin on the expression of vascular endothelial growth factor (VEGF) in human breast cancer cells was examined. Apigenin reduced the secretion of VEGF, mRNA levels of VEGF and the transcription activity of VEGF. Furthermore, the inhibitory effect of apigenin on the transcription activity of VEGF could be reversed by transferring pCEP4-HIF-1alpha into the cells [43]. 

### 2.5. Cell Cycle Arrest

Cancer is a multifactorial factor involving uncontrolled cells. In vivo and in vitro-based studies have confirmed that medicinal plants or their active compounds play a significant role in the cell cycle arrest through G2/M or G0/G1 checkpoint arrest. To measure whether pharmacologic interference with apigenin has a direct growth inhibitory effect on human prostate tumors implanted in athymic nude mice, the cell cycle regulatory molecules were investigated. Apigenin feeding by gavage to these mice was at doses of 20 and 50 microg/mouse/d in 0. The oral intake of apigenin resulted in a dose-dependent increase in the protein expression of KIP1/p27, WAF1/p21, INK4a/p16, as well as INK4c/p18, the down-modulation of the protein expression of cyclins D1, D2, and E; and cyclin-dependent kinases (cdk), cdk2, cdk4, and cdk6 [44]. To recognize the mechanism accountable for apigenin-induced cell growth inhibition and cell cycle progression was evaluated. Apigenin administration generated a concentration-dependent cell cycle arrest in the G2/M phase. The levels of p53 and its downstream protein p21CIP1/WAF1 that is a strong cyclin-dependent kinase (CDK) inhibitor in G1 and G2/M phases has been reported to be increased in a concentration-dependent way when the cells were exposed to apigenin [45]. 

Apigenin was studied for its effects and mechanism of action in renal cell cancer. Apigenin treatment at varying concentrations (5–80 M) inhibited the proliferation of the normal renal proximal tubule epithelial cell line HK-2, ACHN, 786-0, and Caki-1 RCC cells in a dose- and time-dependent manner. Compared to DMSO controls, exposure to apigenin increased comet formation and caused dose-dependent DNA damage. A further indication of double-stranded DNA breaks was a progressive rise in phosphorylated H2AX (γH2AX), as seen by western blotting. Apigenin was discovered to cause damage to DNA, especially at larger dosages, and to trigger cell cycle arrest at the G2/M phase via ATM signal modulation. According to these findings, apigenin administration can cause cell cycle arrest at the G2/M phase, damage to DNA, accumulation of p53, and apoptosis, all of which decrease cancer cell growth in vitro and in vivo [23]. The cellular mechanisms causing the induction of cell cycle arrest by apigenin was examined. Apigenin decreased cell growth and produced cell cycle arrest in the G2/M phase in a breast cancer cell line at the nonapoptotic induction dose. Furthermore, apigenin inhibited the expression of cyclin B, cyclin A, and cyclin-dependent kinase-1, all of which influence the cell cycle G2-to-M phase transition [24]. Apigenin induced apoptosis, cell cycle arrest, ferroptosis and autophagy in NCI-H929 cells. Apigenin may be an appropriate candidate for multiple myeloma treatment. The inhibition of the STAT1/COX-2/iNOS signaling pathway by apigenin is an important mechanism in the suppression of inflammation as well as the induction of apoptosis [46].

### 2.6. PI3K/AKT Pathway

The anti-cancerous role of apigenin was evaluated based on hepatocellular carcinoma cells. Apigenin suppressed cell growth and promoted the death of cancer cells in a dose- and time-dependent approach. Furthermore, apigenin plays role in the induction of apoptosis as well as autophagy via the inhibition of PI3K/Akt/mTOR pathway [25]. Apigenin or apigenin plus Glucose transporter-1 (GLUT-1) AS-ODNs improved the radiosensitivity of xenografts. Apigenin or apigenin plus GLUT-1 decreased the expression of GLUT-1, Akt, and PI3K mRNA after X-ray radiation. Moreover, the effects of apigenin on inhibiting xenograft growth and enhancing xenograft radiosensitivity might be linked with suppressing the expression of GLUT-1 via the PI3K/Akt pathway [47]. Adriamycin caused an increase in the serum levels of cardiac injury markers and enhanced cardiomyocyte apoptosis and autophagy. Apigenin administration prevented the effects associated with Adriamycin -induced cardiotoxicity in mice and inhibited ADR-induced apoptosis and autophagy. API also promoted PI3K/AKT/mTOR pathway activity in ADR-treated mice [48]. Prostate tumorigenesis in transgenic adenocarcinoma of the mice prostate was reported to be suppressed by apigenin. Apigenin-treated mice had lower FoxO3a (Ser253) and Akt (Ser473) and phosphorylation that was linked to higher nuclear retention and reduced FoxO3a binding [26]. 

### 2.7. NF-κB Signaling

Apigenin suppressed prostate cancer in TRAMP rats by interfering with NFκ-B activation in microgram dosages. Apigenin administration to TRAMP mice resulted in a significant reduction in prostate tumour sizes and the complete elimination of metastasis, which was linked to the suppression of DNA binding and NFκ-B activation. Furthermore, after apigenin feeding, NFκ-B-regulated gene products expression implicated in proliferation (COX-2 as well as cyclin D1), anti-apoptosis, and angiogenesis was downregulated [27]. 

Apigenin binds to IKK directly, inhibiting IKK kinase activity and suppressing NF-B/p65 activation in human prostate cancer cells far more efficiently than an IKK inhibitor. Apigenin was also shown to trigger cell cycle arrest in prostate cancer cells, similar to knocking down IKK. Moreover, the inhibition of cell proliferation, invasiveness and decrease in tumor growth by apigenin are mediated by its ability to suppress IKKα and downstream targets affecting NF-ĸB signaling pathways [26]. 

The interactions between apigenin and TRAIL in non-small cell lung cancer cells was determined. The synergistic effect between apigenin and TRAIL on the apoptosis of cancer cells was observed. In the meantime, apigenin suppressed NF-κB, ERK, and AKT activation. Furthermore, apigenin inhibited the prosurvival regulators NF-B, ERK and AKT, resulting in antitumor action in lung cancer cells stimulated by TRAIL [28].

### 2.8. MAPK/ERK Signaling

Apigenin has been reported to cause apoptosis and reduce the ANA-1 cells’ viability in a dose- and time-dependent fashion. Apigenin also decreased phospho-JNK and phospho-ERK expression, increased phospho-p38 expression, and caused cell death in mouse macrophage ANA-1 cells, perhaps via boosting intracellular reactive oxygen species, regulating the MAPK pathway, and subsequently restricting Bcl-2 expression. [49]. Apigenin is a flavonoid produced from plants that has been shown to have anticancer properties in vitro and in vivo studies. Apigenin has been shown to interact with colon cancer cells. For one day, HCT116 cells were exposed to various apigenin doses with or without a constant concentration of ABT-263. While only a small amount of cell death (about 15% with either 20–30 µM of apigenin or 1 µM of ABT–263) was observed when the cells were exposed to apigenin or ABT–263 alone, there was a significant increase in death rate (up to 80%) when the two agents were combined. Apigenin caused cell death in a dose-dependent manner in the presence of 1 µM ABT-263. ERK and AKT activation were shown to be inhibited by apigenin. Furthermore, inhibiting the Mcl-1, ERK and AKT, and prosurvival regulators with apigenin enhances ABT-263-induced antitumor activity in colon cancer cells [29]. 

The effect of apigenin on IGF signaling and its downstream targets in TRAMP mice was evaluated. The result revealed that the administration of apigenin caused a significant reduction in the levels of IGF-I and an increase in the levels of IGFBP-3 and the dorso-lateral prostate. This modulation of IGF/IGFBP-3 was linked with an inhibition of p-ERK1/2 and p-Akt and [50]. The anti-cancer effects of apigenin on choriocarcinoma cells was investigated. Apigenin pretreatment with JAR and JEG3 cells lowered the phosphorylation of AKT, S6 and P70RSK, while increasing the phosphorylation of P90RSK and ERK1/2 in a dose-dependent manner. Apigenin is also a potent chemo preventive drug that suppresses choriocarcinoma cell development and metastasis via regulating the PI3K/AKT and ERK1/2 MAPK signalling transduction pathways [30].

### 2.9. Signal Transducer and Activator of Transcription 3 (STAT3)

STAT3 is indeed an oncogene that promotes cancer cell proliferation, motility, cell survival, and progression [51]. Aberrant activation of STAT3 in cancer cells causes the continuous transcription of cell growth factors and anti-apoptotic molecules that play a crucial role in maintaining cell growth and survival [52].

Apigenin shows a vital task in cancer management by decreasing the activity of the Signal Transducer and the Activator of Transcription. Apigenin promoted cell death and autophagy in primary effusion lymphoma (PEL) cells, as well as a decrease in intracellular reactive oxygen species, according to Marisa Granato et al., 2017. In addition, apigenin activated p53, which led to the induction of catalase, a reactive oxygen species scavenger enzyme, and the inhibition of Signal Transducer and Activator of Transcription 3, the most critical pro-survival pathway in primary effusion lymphoma. Additionally, apigenin has been shown to suppress STAT3 that resulted in p53 activation [31].

Another study result reported that apigenin binds to non-phosphorylated signal transducer and activator of Transcription 3, decreases the signal transducer and activator of the Transcription 3 phosphorylation and transcriptional activity in VAT, and thus decreases the expression of the signal transducer and activator of the Transcription 3 target gene cluster of differentiation 36 [53]. Apigenin effectively inhibited Signal Transducer and Activator of Transcription 3 phosphorylation, lowered STAT3 nuclear localization, and suppressed Signal Transducer and Activator of Transcription 3 transcriptional activity, according to another noteworthy discovery. Apigenin also inhibited the Signal Transducer and Activator of Transcription 3 target genes of matrix metalloproteinase-2, matrix metalloproteinase-9, and vascular endothelial growth factor, which are involved in cell migration and invasion [32].

### 2.10. Epidermal Growth Factor Receptor (EGFR)

Apigenin and Cetuximab, a recognized epidermal growth factor receptor inhibitor, were studied in conjunction with the treatment of nasopharyngeal cancer in a significant study. Results demonstrated that it produced a greater pro-apoptosis effect. Apigenin and Cetuximab, on the other hand, have been shown to reduce the expression of p-STAT3, p-Akt, p-EGFR, and Cyclin D1 [33]. The combination of apigenin and gefitinib on epidermal growth factor receptor (EGFR)-resistant mutant non-small cell lung cancer was evaluated. It was noticed that apigenin combined with gefitinib inhibits multiple oncogenic drivers including HIF-1α, EGFR, c-Myc, and reduces MCT1 and Gluts protein expression, Consequently, the combined apigenin + gefitinib treatment grants an attractive strategy as an alternative treatment for the acquired resistance to epidermal growth factor receptor -TKIs in lung cancer [54].

### 2.11. Wnt/β-Catenin Pathway

Apigenin was found to have anti-cancer action, as it prevented colorectal cancer cell proliferation, migration, as well as invasion in a concentration-dependent pattern. Furthermore, by suppressing the Wnt/-catenin signalling pathway, apigenin greatly reduced colorectal cancer cell proliferation, migration, invasion, as well as organoid growth [34]. A study was undertaken to evaluate the role of apigenin as an anti-cancer agent, and apigenin has been shown to suppress human osteosarcoma cell growth and invasion. Apigenin suppresses osteosarcoma cell tumour development by inactivating Wnt/-catenin signalling [55]. 

## 3. Apigenin: Inhibition and Prevention of Various Types of Cancer

**Figure 3:**
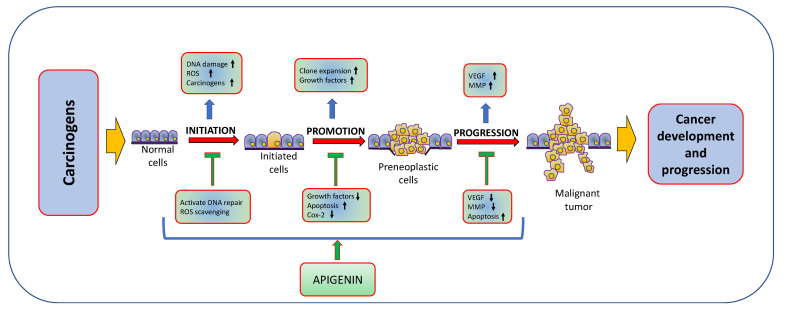
Anti-cancer activity of apigenin: Apigenin plays a significant role in cancer inhibition through inhibiting the carcinogenesis by many various molecular interactions and processes, such as the regulation of the apoptotic machinery, aberrant cell signaling and oncogenic protein network.

**Figure 4:**
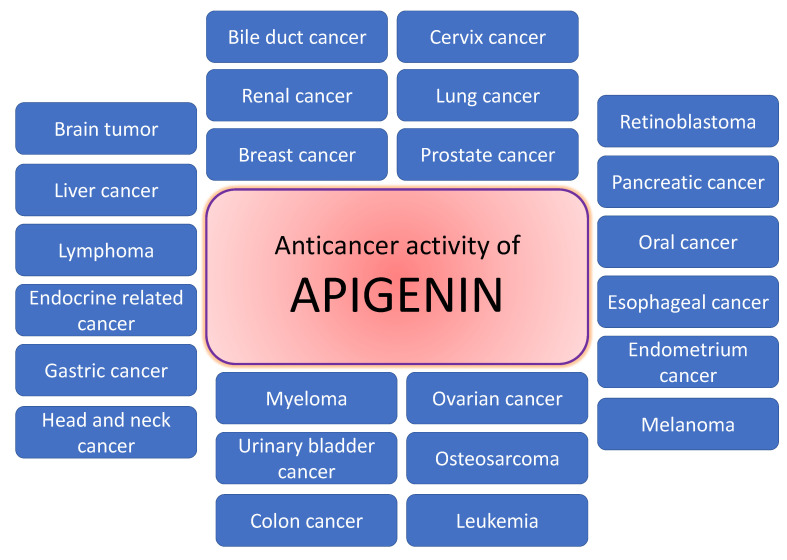
Protective effect of apigenin against different cancer types. Apigenin has been widely investigated for its anti-cancer activities and low toxicity. Apigenin has been shown to inhibit a number of human cancers both in vitro and in vivo through a variety of biological mechanisms.

Natural products or active compounds of medicinal plants show a role in cancer management through modulating various biological activities [56,57,58]. In this regard, in vivo and in vitro studies have proven that apigenin plays a significant role in cancer management through modulating cell signalling pathways and the process of carcinogenesis (Figure 3). The role of apigenin in cancer inhibition and treatment of various cancers are described in Table 2 and Figure 4;

### 3.1. Breast Cancer

The effects of apigenin on estrogen-responsive, antiestrogen-sensitive MCF7 breast cancer cells and two MCF7 sublines with acquired resistance to either fulvestrant or tamoxifen was examined. Results revealed that apigenin functions as both an estrogen and antiestrogen, in a dose-dependent fashion. Moreover, at high concentrations of apigenin, the inhibited growth of cancer cells and the antiestrogen-resistant sublines, and the combination of apigenin with either tamoxifen or fulvestrant demonstrated synergistic, growth-inhibitory effects. Finally, the results showed that apigenin, via its capability to target both ERα-dependent and independent pathways, has potential as a new therapeutic agent against antiestrogen-resistant breast cancer [99]. 

Another experiment was performed to examine the human breast cancer cell lines having different levels of expression of neu/HER2 and to establish that apigenin exhibited potent growth-inhibitory activity in HER2/neu-overexpressing breast cancer cells. Moreover, the induction of apoptosis was also seen in neu/HER2-overexpressing breast cancer cells in a dose- and time-dependent way. Moreover, apigenin inhibits Akt function in tumor cells in a complex manner. Apigenin straight inhibited the activity of PI3K whereas indirectly inhibiting the activity of Akt kinase as well as the inhibition of HER2/neu autophosphorylation and transphosphorylation resulting from depleting HER2/neu protein in vivo was also noticed [59]. 

The mechanistic role of the caspase cascade in extrinsic and intrinsic apoptosis induced by apigenin, as development of noncytotoxic anticancer medicines was investigated. Treatment with apigenin (1–100 µM) meaningfully inhibited the proliferation of human breast cancer cells in a dose and time-dependent fashion. In cells exposed to apigenin, this inhibition resulted in the activation of apoptosis and the discharge of cytochrome c. Apigenin then cleaved caspase-9, which is involved in mitochondria-mediated apoptosis. Furthermore, apigenin activated caspase-3, which works downstream of caspase-9, and caspase-3 activation was followed by dissociation of capases-6,7,8 [60]. 

In human breast cancer cells, the effects of 5-fluorouracil and apigenin combination therapy on proliferation and apoptosis were investigated. Breast cancer cells that have been exposed to ErbB2 overexpression were resistant to 5-fluorouracil. Surprisingly, co-administration with apigenin considerably reduced resistance. Cellular proliferation was drastically lowered by 5-fluorouracil with apigenin when compared to 5-fluorouracil alone. Overall, the findings suggest that 5-fluorouracil and apigenin work synergistically to limit cell growth and induce apoptosis via Akt activation and ErbB2 expression [61].

### 3.2. Cervical Cancer

Apigenin has a specific cytotoxic action and it may trigger apoptosis in all cervical cancer cell lines, according to a key study result. Apigenin was also found to cause mitochondrial redox impairment by increasing reactive oxygen species and H2O2, decreasing the mitochondrial membrane potential, and increasing Lipid peroxidation levels. As a result, apigenin appears to be a promising novel anticancer treatment candidate for cervical cancer caused by several human papillomavirus genotypes [100]. Apigenin was tested in a landmark study to see if it may lessen the influence of histamine on tumours by modulating the expression level of oestrogen receptors, thereby inhibiting cervical cancer progression. In order to test the hypothesis that apigenin inhibited HeLa cell proliferation by changing the ER signal, the mRNA levels of the genes encoding ERα and ERβ were measured in the cells. It is interesting to note that apigenin significantly altered the expression of the ERα and ERβ genes. Apigenin suppressed cell growth in human cervical cancer cells in a dose-dependent fashion, according to in vitro findings. Furthermore, the xenograft model was utilized to investigate the anticancer properties of apigenin in vivo, and the findings revealed that apigenin prevented cervical tumour development by reverting the aberrant ER signal in tumour tissue induced by histamine [101]. 

Apigenin inhibited the growth of human cervical carcinoma cells through the apoptotic pathway. Furthermore, apigenin significantly decreased the viability of cancer cells, and apigenin-induced apoptosis in cervical cancer cells was confirmed and induction of sub-G1 phase. Apigenin-treated cancer cells became arrested in the G1 phase that was linked to a significant increase in the expression of the p21/WAF1 protein. WAF1/p21 induction appears to be transcriptionally accelerated and p53 dependent [62], and other findings reported anti-cancerous activity [102]. Analysis of two sub-clones of HeLa cells including low invasive potential HeLa wt cells and their Cx43 transfected counterparts allowed for the pinpointing of the main effects of apigenin on invasion and to identify the significance of cell proliferation and motility during invasive processes. A distinct inhibition of cell movements rather than a reduction in cell proliferation was associated with a decrease in the invasive potential of HeLa Cx43 cells after treatment with apigenin, especially at low apigenin concentrations. The effect of apigenin on cell proliferation was less marked, particularly at low apigenin concentration, while its effect on cell motility linked with the decrease of the invasive potential of HeLa Cx43. Furthermore, apigenin inhibitory impact on tumour cell invasiveness in vitro suggests that it may conduct its anti-tumorigenic activity in vivo by inhibiting tumour cells from penetrating healthy tissue [63].

### 3.3. Ovarian Cancer

Apigenin as a dietary flavonoid, has been revealed to hold anti-tumor properties. Apigenin inhibits the proliferation and tumorigenesis of human ovarian cancer cells through the inhibition of the differentiation or DNA binding protein 1 (Id1). Apigenin inhibited the expression of Id1 by promoting transcription factor [64]. Another ovarian cancer study found that apigenin produced early apoptosis in 24 h, whereas α-mangostin and doxorubicin triggered late apoptosis and necrosis after 72 h. Additionally, in apigenin-treated cancer cells, caspase-9 activity was dramatically elevated at 24 h, and both α-mangostin and apigenin interrupted the cell cycle in the G2/M phase [65]. 

Another finding suggests that apigenin inhibited the self-renewal capacity of SKOV3-derived SFCs and was involved in downregulating the expression of Gli1 by the inhibition of CK2α [66]. Yuyan Qi et al., 2020 tested the effects of apigenin on cell death and resistance to cisplatin in ovarian cancer cells. Apigenin decreased proliferation, slowed cell cycle progression, and induced apoptosis in cancer cells according to the findings. These effects were also detected in SKOV3/DDP cells that were resistant to cisplatin. Moreover, apigenin decreased mitochondrial transmembrane potential and increased the ratios of cleaved caspase-3/caspase-3 and Bax/Bcl-2 in both tested cell lines. The findings reveal the role of apigenin in ovarian cancer cell death and resistance to cisplatin, as well as the molecular pathways involved [103]. 

Another study based on ovarian cancer reported that apigenin decelerated ovarian cancer development via downregulating ER-mediated PI3K/AKT/mTOR expression, therefore demonstrating its applicability as a potentially effective therapeutic agent for ovarian cancer treatment [104]. In comparison with the control group, apigenin dramatically reduced Matrix metallopeptidase 9 expression as well as p-AKT and p-p70S6K1 levels in cancerous tissue. The AKT/p70S6K1 pathway was also involved in the downregulation of Matrix metallopeptidase 9 by apigenin. Furthermore, it was reported that the oral uptake of apigenin can inhibit tumor metastasis through Matrix metallopeptidase 9 expression using the orthotopic ovarian tumor model [105].

### 3.4. Pancreatic Cancer

Apigenin cytotoxic impact on two pancreatic cell lines was studied in order to see if it might prevent various p53 mutations. In comparison to PaCa44, apigenin was shown to have a higher cytotoxic impact on the Panc1 cell line. Furthermore, apigenin cytotoxicity was linked to an increase in intracellular reactive oxygen species, a decrease in mutant p53 and HSP90 expression, and mTORC1 suppression [67]. 

Whether apigenin inhibits pancreatic cancer cell growth in vitro was explored. Findings from a study revealed that apigenin caused both the time and concentration-dependent inhibition of DNA synthesis and cell proliferation in four pancreatic cancer cell lines. AsPC-1, CD18, MIA PaCa2, and S2-013 were the four human pancreatic cancer cell lines. In all four cell lines examined, apigenin significantly reduced DNA synthesis in a concentration-dependent manner. The proportion of cells in the G2/M phase rises in tandem with higher apigenin concentrations. When compared to control, a decrease in cell number over a 72-h period was associated with the inhibition of DNA synthesis. In almost every cell line, the number of cells increased in untreated cells every 24 h, whereas it increased more slowly or decreased in apigenin-treated cells. Additionally, apigenin induced G2/M phase cell cycle arrest and reduced levels of cyclin A, cyclin B, phosphorylated forms of cdc25 and cdc2 [68]. In pancreatic cancer cell lines, the effects of apigenin on geminin expression and other replication proteins were studied. Apigenin downregulates geminin expression at both the mRNA and protein levels, according to real-time RT-PCR and western blotting analyses. The application of cells with the proteosome inhibitor MG132 reversed apigenin downregulation of geminin, indicating that the degradation route is another way apigenin influences geminin expression. Overall, the findings revealed that geminin was overexpressed in pancreatic cancer and was downregulated by apigenin, possibly adding to apigenin anticancer impact [106]. In pancreatic cancer cells, the involvement of nuclear factor-κB in apigenin-induced growth suppression was studied. Apigenin inhibited cell development and caused apoptosis in the cells, according to the study. Apigenin administration also reduced both baseline and TNF-α-induced NF-κB DNA binding activity as well as NF-B transcription activity. Additionally, apigenin had the potential to inhibit IKK-β-mediated nuclear factor-κB activation, and was a valued agent for pancreatic cancer treatment [69]. In human pancreatic cancer cells, the flavonoid apigenin was tested for its effects on glucose uptake, glucose transporter 1 (GLUT-1) expression, and the phosphoinositide 3-kinase (PI3K)/Akt pathway. Apigenin effectively decreased the uptake of glucose in CD18 and S2-013 human pancreatic cancer cell lines in a dose-dependent way, according to the findings. Both glucose transporter 1 mRNA and protein expression were reduced by apigenin in a dose and time-dependent manner [107]. 

### 3.5. Gastric Cancer

In Mongolian gerbils, the effects of apigenin therapy on Helicobacter pylori-induced atrophic gastritis and stomach cancer development were studied. Results revealed that apigenin treatments effectively decreased the rates of atrophic gastritis and dysplasia/gastritis in Mongolian gerbils. Furthermore, in both atrophic gastritis and gastric cancer, Helicobacter pylori colonization and Helicobacter pylori-induced histological alterations of neutrophil and monocyte infiltrations as well as atrophic gastritis in both Mongolian gerbils gastric cancer and atrophic gastritis was decreased by apigenin therapy [108]. Apigenin treatments meaningfully increased IκBα expression, and therefore inhibited nuclear factor kappa B activation, and the inflammatory factor expressions decreased. The reactive oxygen species levels decreased partly because of the intrinsic scavenging property of apigenin. Moreover, apigenin treatments effectively inhibited NF-κB activation and the related inflammatory factor expressions, as well as increased mucin-2 expression in the H. pylori-infected MKN45 cells [109]. 

### 3.6. Bile Duct Cancer

Apigenin effects on cholangiocarcinoma growth suppression and apoptosis were investigated in a significant study. Apigenin encouraged cancer cells to die by inducing apoptosis. Surprisingly, two proteins, heterogeneous nuclear ribonucleoprotein H and A2/B1, vanished entirely after treatment, suggesting that apigenin plays a role in cell death. Apigenin induces growth inhibition and apoptosis in cholangiocarcinoma cells, and the apoptosis pathway was verified by proteomic analysis [70].

### 3.7. Colon Cancer

Apigenin’s anticancer effect was investigated against cisplatin-resistant colon cancer cells. Apigenin was discovered to substantially suppress the multiplication of colon cancer cells. Apigenin’s anticancer action against colon cancer cells was revealed to be attributable to the stimulation of autophagy as well as apoptosis. Apigenin has also been shown to suppress the m-TOR/PI3K/AKT signalling cascade in colon cancer cells resistant to cisplatin. Moreover, an in vivo based result found that apigenin inhibited the growth of xenografted tumors [71]. 

The outcomes of another study demonstrated that apigenin can inhibit HCT116 and LOVO proliferation in a dose-dependent manner. In comparison to the control groups, the wound-healing experiments demonstrated that apigenin at concentrations of 10 and 20 µM could inhibit the migration of HCT-116 and LOVO cells. Through the NF-kB/Snail signalling pathway, apigenin prevented the EMT of human colon cancer cell lines HCT-116 and LOVO. Thus, apigenin might inhibit the epithelial-mesenchymal transition, migration, and invasion of human colon cancer cells based on in vitro and in vivo via the NF-κB/Snail pathway [72]. Apigenin played a role in the reduction of the proliferation of colon cancer cell lines, stimulated the cleavage of PARP and induced apoptosis in a dose-dependent way. Moreover, apigenin treatment decreased the expression of the anti-apoptotic proteins Bcl-xL and Mcl-1. 

Apigenin also inhibits the anti-apoptotic proteins Mcl-1 and Bcl-xL, causing colon cancer cells to die by blocking the phosphorylation of Signal transducer and activator of transcription 3 and therefore inducing apoptosis [73]. Apigenin has the potential to inhibit cellular glycolysis by inhibiting tumor-specific pyruvate kinase M2 activity and expression while also having anti-colon cancer properties. Furthermore, Apigenin might assure a low PKM2/PKM1 ratio in cancer cells by inhibiting the β-catenin/c-Myc/PTBP1 signal cascade, according to RT-PCR as well as western blot experiments [110]. 

The role of autophagy in the apoptosis of human colon cancer cells caused by apigenin was investigated. Apigenin was found to inhibit cell proliferation in colon cancer cells in a concentration-dependent fashion. Apigenin also caused G2/M phase arrest and inhibited the production of cyclin B1 and its activating partners, Cdc2 and Cdc25c [45]. The role of the adenomatous polyposis coli (APC) tumour suppressor mutation in apigenin potential to cause cell cycle arrest was investigated in another study. Apigenin administration led to a drop in cell number coincident with flow cytometry evidence indicating a dose-dependent increase of cells in the G2/M phase for both cancer cells. When HT29- adenomatous polyposis coli cells were fed with 80 µM apigenin for 120 h, flow cytometric analysis revealed a significant increase in the fraction of cells in G2/M. Additionally, results advocate that adenomatous polyposis dysfunction may be critical for apigenin to induce cell cycle arrest in human colon cancer cell lines [111]. 

### 3.8. Bladder Cancer

The effect and mechanism of apigenin in the human bladder cancer cell line was investigated. The results of the study confirmed that apigenin decreased proliferation and inhibited the migration and invasion potential of bladder cancer cells in a dose- and time-dependent way that was linked with induced G2/M Phase cell cycle arrest as well as apoptosis. Moreover, apigenin increased caspase-3 activity and PARP cleavage, demonstrating that apigenin induced apoptosis in a caspase-dependent way [74]. Interlukin-1β could significantly induce uPAR expression in bladder cancer cells, and apigenin-inhibited IL-1β could induce uPAR expression concentration-dependently. Apigenin, via inhibiting the ERK1/2 and JNK signalling pathways, decreased the transcriptional activity of both Activator protein 1 and NF-κB. These findings suggest that apigenin can reduce uPAR expression via modulating the (ERK1/2, JNK)/Activator protein 1 and (ERK1/2, JNK)/NF-κB signalling pathways in human bladder cancer cells [75].

Another study based on bladder cancer reported that apigenin inhibited cancer cell proliferation in a dose-dependent manner. Moreover, apigenin-induced early and mid-apoptotic cells and induce the loss of the mitochondrial membrane potential. Finally, results indicated that apigenin inhibits cancer cells proliferation through blocking cell cycle progression and inducing apoptosis [112]. Apigenin significantly inhibited MAPK activation/phosphorylation and the migration of hBSM cells triggered by MEKK1 overexpression. Furthermore, apigenin hindered actin polymerization, which is important for muscle contraction and cell migration. The results propose that apigenin inhibits the activation of MAPKs and thus the cell migration [76]. 

### 3.9. Liver Cancer

Treatment with apigenin and chrysin reduced liver cancer HepG2 and breast cancer MDA-MB-231 cell viability as well as induced apoptosis via down-regulation of S-phase kinase-associated protein-2 (Skp2) and low-density lipoprotein receptor-related protein 6 (LRP6) expression. The experimental results showed that chrysin combined with apigenin can reduce liver cancer HepG2 and breast cancer MDA-MB-231 proliferation and cell motility and induce apoptosis [113].

The role of apigenin as an anticancer agent was further evaluated against hepatocellular carcinoma cells. The study indicated that apigenin, in a dose- and time-dependent way, suppressed cell proliferation and promoted cell death. Apigenin also raised the expression of LC3-II and the density of GFP-LC3 puncta. Furthermore, apigenin triggered apoptosis and autophagy by inhibiting the PI3K/Akt/mTOR pathway. Additionally, in vivo results demonstrated that apigenin treatment reduced tumour growth and autophagy suppression by 3-MA became significantly enhanced due to apigenin anticancer effect [25]. Apigenin induced G1 arrest in HepG2 in a dose-dependent manner. CyclinD1 was up-regulated and CDK4 was down-regulated upon Apigenin treatment that designated that Apigenin might block cell cycle progression at the G1 phase via the regulation of CDK4 as well as CyclinD1 expression [77]. Another study looked at the synergetic therapeutic benefits of sorafenib and apigenin on apoptosis and the cell cycle. The combined therapy of sorafenib and apigenin had a greater decrease in cell viability than the solo treatment groups. Furthermore, the combination sample resulted in a considerable increase in apoptotic cells. Cell migration and invasion abilities were reduced in the combined therapy group. The combined treatment results on migration, invasion, apoptosis, and gene expression revealed that sorafenib with apigenin may have a synergistic impact [114]. Apigenin potential chemo-sensitization activity in doxorubicin-resistant hepatocellular carcinoma cell line was investigated. Apigenin was shown to significantly increase the sensitivity of doxorubicin, stimulate expression of miR-520b, and suppress ATG7-dependent autophagy. Furthermore, miR-520b mimics improved doxorubicin sensitivity while inhibiting ATG7-dependent autophagy [78]. Apigenin decreased proliferation, migration, and invasion in PLC cell xenografts through a dose-dependent approach, suppressing tumour development without changing body weight and thereby extending the lifespan. Apigenin also decreased Snai1 and Nuclear factor-κB expression, overturned increases in epithelial-mesenchymal transition marker levels, promoted cellular adhesion, influenced actin polymerization and cell migration, and suppressed the invasion and migration of hepatocellular carcinoma cells [115]. Apigenin could enhance the cytotoxicity of 5-fluorouracil in hepatocellular cancer by inhibiting reactive oxygen species-mediated drug resistance and simultaneously activating mitochondrial apoptosis pathways [116].

### 3.10. Renal Cancer

Apigenin suppressed renal cell carcinoma cell proliferation in a dose- and time-dependent manner. Apigenin also produced damage to DNA in ACHN cells, especially at greater dosages, and promoted cell cycle arrest in the G2/M phase via ATM signal modulation. In addition, in vivo, apigenin therapy decreased tumour growth and volume. Finally, apigenin treatment causes damage to DNA, cell cycle arrest in the G2/M phase, p53 accumulation, and apoptosis [23]. Another study result revealed that the level of lipid peroxidation significantly increased in carcinogen administered animals, and this reverted almost back to normal by apigenin treatment. Moreover, the activities/levels of the antioxidant status both in liver and kidney were decreased in carcinogen administered animals, which was brought back to near normal upon apigenin administration. Finding advocates that apigenin prevents lipid peroxidation and protects antioxidant system in N-nitrosodiethylamine induced and phenobarbital promoted hepatocellular carcinogenesis [79].

### 3.11. Esophageal Cancer

In human esophageal cancer cells, apigenin was found to suppress interlukin-6 transcription and gene expression. Additionally, apigenin meaningfully and dose-dependently inhibited cell proliferation and promoted apoptosis and suppressed expression of VEGF and tumor-induced angiogenesis. It was reported that apigenin-induced cellular modifications might be totally reversed by the pre-treatment of cells with interlukin-6. Eventually it was discovered that apigenin is a novel inhibitor of interlukin-6 transcription and that blocking interlukin-6 transcription is amongst the ways through which apigenin exerts its anticancer effects [117]. Another analysis revealed that apigenin enhanced membrane permeability and produced LDH leakage that seemed steady with AFM-detected damage to the membrane ultrastructure. As a result, membrane ultrastructure damage and increased membrane permeability figured prominently in apigenin-induced human oesophageal cancer cell apoptosis [80].

### 3.12. Head and Neck Cancer

The apigenin dose-dependently inhibits survival and induces the apoptosis of head and neck squamous cell carcinoma cells. Furthermore, results advocate that apigenin properties might be exploited for chemoprevention and/or therapy of head and neck carcinomas [118]. The effect of apigenin on head and neck squamous cell carcinoma cells was examined. The viability of HN-30 cells was significantly decreased by the application of apigenin in a dose- and time-dependent fashion. Apigenin also drastically reduced the mRNA expression of NANOG, CD44 and CD105. Apigenin suppresses cancer stem cell marker expression and the number of cells expressing cell surface markers under hypoxia [81]. The effect of apigenin and kaempferol on cell viability in cultured cells derived from the pharynx, an oral cavity carcinoma, and a metastatic lymph node and in explanted FaDu cells was investigated. The FaDu cell line was used to assess how apigenin and kaempferol affected the viability of HHNSCC cells in vitro. Treatments with apigenin and kaempferol reduced cell viability in a dose-dependent manner. Kaempferol was more effective than apigenin at lowering cell viability at the lower doses. Apigenin (half maximal inhibitory concentration) was more efficient than kaempferol at reducing cell viability at higher doses and after 48 h of treatment. Apigenin and kaempferol treatment decreased viability of cells in vitro, and cell-type-dependent differences in responsiveness were observed. In vivo apigenin treatment significantly increased the tumor size of FaDu explants [82]. Another study result showed that apigenin reduced the proliferation of human nasopharyngeal carcinoma cells triggered through C5a through negative regulation of the C5aR/PCAF/STAT3 axis [119].

### 3.13. Lung Cancer

An experiment was performed to evaluate whether apigenin improved the antitumor efficiency of cisplatin in lung cancer. CD133 positive cells were found to be inhibited by apigenin, and cisplatin’s antitumor effect in lung cancer cells also became improved by apigenin. The p53 inhibitor Pifithrin-, as well as siRNA targeting the p53 gene, were used to disrupt the synergistic antitumor activity of Apigenin and cisplatin in A549R cells. However, apigenin inhibits cisplatin-induced CSC via p53, as A549R cells without p53 and Pifithrin-treatment reduced the drop in CD 133 positive cells following apigenin administration in cisplatin-treated lung cancer cells [120]. The lung cancer H460 cells were treated with apigenin and resulted in the dose-dependent induction of morphological changes and induced DNA damage and apoptosis; the down-modulation of the protein expression of Bid, Bcl-2, and the up-regulation of protein levels of Bax decrease in the percentage of viability, caspase-3, and increased the productions of reactive oxygen species in lung cancer cells [121]. The synergistic effect between apigenin and TRAIL on the apoptosis of non-small cell lung cancer cells was observed. Apigenin enabled non-cell lung cancer cells more susceptible to TRAIL-induced apoptosis by increased expression of the levels of death receptor 4 and death receptor 5 in a p53-dependent way. Bcl-xl and Bcl-2, anti-apoptotic proteins, were consistently significantly suppressed, whereas Bad and Bax, pro-apoptotic proteins, were increased [28]. Both apigenin and luteolin significantly suppressed lung cancer with KRAS mutant proliferation, and down-regulated the IFN-γ induced PD-L1 expression [122].

### 3.14. Oral Cancer

Apigenin therapy caused cell cycle arrest at both the G0/G1 and G2/M checkpoints, as well as CDK1 inactivation and reduced levels of cyclin D1 and E. These findings demonstrate apigenin anticancer capability for an oral squamous cell carcinoma cell line, implying that it could be a promising chemo preventive drug due to its cancer cell cytotoxic ability and potential to serve as a cell cycle modifying therapy at multiple levels [83].

### 3.15. Lymphoma

Apigenin, alone or in combination with abivertinib, was tested for its ability to suppress DLBCL progression, the most frequent kind of aggressive lymphoma advancement. The outcomes show the expression of caspase family proteins, including cleaved-PARP, cleaved-C3, and cleaved-C8 in a dose-dependent manner. Apigenin caused apoptosis in diffuse large B cells by influencing both mitochondrial and caspase family apoptotic pathways, and in diffuse large B cells, apigenin disrupts the cell cycle. Analysis of the flow cytometry data revealed that apigenin could significantly arrest the G2/M phase in U2932 and LY10 at a very low dose, suggesting it to be the primary mechanism of action of apigenin in diffuse large B-cell lymphoma. The findings indicated the downregulation of CDK2, CDK4, CDK6, Rb, and CDC. Apigenin inhibited DLBCL cell proliferation and clone formation while inducing apoptosis through down-regulating BCL-XL and activating the Caspase family, according to the findings. Furthermore, apigenin inhibits DLBCL cell viability by significantly reducing the expression of the pro-proliferative pathway PI3K/mTOR. In addition, in vitro and in vivo data demonstrate that apigenin can synergistically induce apoptosis with abivertinib, a new BTK inhibitor, in the treatment of DLBCL [84]. Apigenin caused primary effusion lymphoma cell death and autophagy, as well as a decrease in intracellular reactive oxygen species, according to Marisa Granato et al. Furthermore, as indicated by p53 silencing, apigenin stimulated p53, which promoted catalase, and blocked the signal transducer and activator of transcription 3, the most critical pro-survival pathway in primary effusion lymphoma [52]. 

### 3.16. Melanoma

The anti-cancer effect of apigenin on human melanoma was examined. In vitro investigation verified that the proliferation of melanoma cells was efficiently inhibited by Apigenin. A375 and C8161 cells were exposed to various concentrations (40, 80, 120, 160, 200, 240, and 280 M) of apigenin for various amounts of time in order to investigate the growth-inhibitory effect of apigenin (24, 48, 72, and 96 h). The MTT assay was used to determine the cell viability. The relatively strong dose- and time-dependent inhibition of cell growth caused by apigenin was evident. Furthermore, it inhibited cell invasion and migration and the induction of G2/M phase arrest as well as apoptosis. Also, apigenin promoted the cleaved PARP proteins and activation of cleaved caspase-3 and decreased the expression of phosphorylated (p)-ERK1/2 proteins, p-AKT and p-mTOR [85]. Polyphenols were tested in vivo for their impact on melanoma cell proliferation and metastatic potential. At the time of i.m. injection of melanoma cells into syngeneic mice, intraperitoneal administration of quercetin, apigenin, (-)-epigallocathechin-3-gallate, resveratrol, and the anti-estrogen tamoxifen led to a substantial, dose-dependent delay in tumor formation. Moreover, in a dose-dependent way, quercetin, apigenin, and tamoxifen dramatically reduced the quantity of B16-BL6 colonies in the lungs, while quercetin and apigenin became more efficacious with regard to tamoxifen [123].

### 3.17. Malignant Mesothelioma

Apigenin anti-tumoral properties in malignant mesothelioma were studied in vitro and in vivo. The effect of apigenin on the development and demise of malignant mesothelioma (MM) cell lines. The SRB assay was used to measure the growth of human (MM-B1, H-Meso-1, MM-F1) and mouse MM cell lines after 24, 48, and 72 h of DMSO or Apigenin treatment. The DCF-DA assay was run on API-treated MM cells to see how the API affected the production of intracellular ROS. When ROS damaged DNA, the histone H2AX variant (H2AX) was quickly phosphorylated at Ser 139 (-H2AX). Apigenin decreased the viability of malignant mesothelioma cells in vitro, increased the intracellular synthesis of reactive oxygen species, and caused damage to DNA. The rise of the Bax/Bcl-2 ratio, the increase of p53 expression, and the activation of both caspase 9 and caspase 8 all contributed to the continuation of API-induced apoptosis. The intraperitoneal administration of apigenin increased the median survival of mice and reduced the risk of tumor growth [86]. 

### 3.18. Adenoid Cystic Carcinoma

Apigenin is a natural flavonoid which causes various biological effects, including anticancer activities. Apigenin was tested to see if it decreases the growth of adenoid cystic carcinoma cells or lowers the expression of GLUT-1 in these cells. Apigenin suppresses adenoid cystic carcinoma-2 cell proliferation in a dose- and time-dependent manner according to the findings. Apoptosis and G2/M-phase arrest was also triggered by apigenin treatment in a dose- and time-dependent way [87].

### 3.19. Myeloma

The role of apigenin on multiple myeloma cell lines, and on primary multiple myeloma cells, was investigated. Apigenin cytotoxicity was established in cell viability experiments. Furthermore, the therapy reduced the expression of antiapoptotic proteins and survivin, resulting in the apoptotic cell death in multiple myeloma cells. In fact, when apigenin was combined with the Hsp90 inhibitor geldanamycin and the histone deacetylase inhibitor vorinostat, it was successful in lowering Hsp90 clients [88]. In serum-starved neuroblastoma cells, a combination of synthetic retinoid N-(4-hydroxyphenyl) retinamide (4-HPR) and apigenin reduced cell survival. Furthermore, the combined treatment decreased autophagy while increasing autophagy inhibitory p-Akt/mTOR signaling. In malignant neuroblastoma cells, the combination of 4-HPR with apigenin inhibits autophagy and promotes apoptosis in a synergistic manner [124].

### 3.20. Leukemia

With regard to the molecular concerns of apigenin treatment in leukemia, the myeloid and erythroid subtypes was analysed. Apigenin blocked proliferation in both lineages via cell-cycle arrest in the G_2_/M phase for myeloid HL60 and G_0_/G_1_ phase for erythroid TF1 cells. In both cell lines the JAK/STAT pathway was one of the major targets of apigenin. The findings revealed that although apigenin is a potential chemopreventive agent due to the induction of leukemia cell-cycle arrest [89]. The involvement of the Akt and JNK signalling cascades in apigenin-induced apoptosis of human leukaemia cells, as well as the anti-leukemic efficacy of apigenin in vivo, were investigated. Apigenin-induced apoptosis was shown to be triggered by the inactivation of Akt, which is accompanied by the activation of JNK, Mcl-1, and Bcl-2. In addition, blocking the JNK pathway resulted in a significant decrease in apigenin-induced caspase activation and death in leukemia cells. In U937 xenografts, the in vivo treatment of apigenin was shown to inhibit tumor development via Akt inactivation and JNK activation [90]. 

### 3.21. Prostate Cancer

A study based on prostate cancer reported that apigenin-induced apoptosis in 22Rv1 cells is initiated by a reactive oxygen species-dependent disruption of the mitochondrial membrane potential via transcriptional-dependent as well as -independent p53 pathways [125]. A pioneer study was performed to examine the effect and mechanism of apigenin on the movement of prostate cancer cells. Outcomes of the study revealed that apigenin suppressed the proliferation as well as inhibited the migration and invasive potential of the prostate cancer cells in a dose- and time-dependent way, which was linked with epithelial mesenchymal transition [91]. Apigenin suppresses the proliferation of androgen-responsive human prostate cancer cells, providing a mechanistic explanation for this activity. Furthermore, apigenin therapy inhibited cell proliferation, which resulted in a considerable drop in AR protein expression, as well as intracellular and released forms of prostate specific antigen. Apigenin treatment also caused the down-modulation of the constitutive expression of NF-kappaB/p65, and apigenin has strong potential for development as an agent for prevention against prostate cancer [20]. Apigenin treatment of androgen-refractory human prostate cancer cells resulted in a dose-dependent decrease in the molecules c-IAP2, XIAP, c-IAP1 along with survivin. Furthermore, apigenin administration led to a considerable reduction in cell viability and the activation of apoptosis, along with a rise in cytochrome C in a time-dependent manner. Reduced Bcl-2 and Bcl-xL levels, as well as a rise in the active form of the Bax protein, were linked to these apigenin aftereffects [19]. Apigenin as well as doxorubicin dose-dependently inhibited cell survival, and co-administration of both compound meaningfully induced cell death through downregulating Bcl-XL and upregulating the mRNA expression of caspases, Bax and cytochrome c [92].

Apigenin inhibits IKKα kinase activity, possesses anti-invasive and anti-proliferative characteristics, and anticancer efficacy in an investigational tumor setup. Apigenin has also been demonstrated to interact directly with IKKα. It has been shown to suppress IKKα kinase activity and NF-B/p65 activation in human prostate cancer cells considerably far more effectively than PS1145, which is a IKKα inhibitor. In prostate cancer cells, Apigenin also caused cell cycle arrest analogous to IKK knockdown [93]. 

### 3.22. Osteosarcoma

The effects of apigenin on human osteosarcoma cells and clarify that the apigenin-induced apoptosis-associated signals was investigated. The outcome of the study demonstrated that apigenin meaningfully decreased cell viability and efficiently induced apoptosis via the activations of caspase and BAX. Moreover, in nude mice bearing U-2 OS xenograft tumours, apigenin inhibited tumour growth [94]. Apigenin anti-proliferation and anti-invasion action on human osteosarcoma cells was examined, as well as linked to possible molecular pathways involved. Apigenin decreased the expression of β-catenin in human osteosarcoma cells and hindered proliferation and invasion. In addition, overexpression of β-catenin abolished apigenin inhibitory action onto osteosarcoma cells. Apigenin suppresses osteosarcoma cell tumour growth by inactivating Wnt/β -catenin signalling, according to the studies [55]. Apigenin inhibited the phosphorylation of PI3K, mTOR Akt, and in SOSP-9607 cells. Together, these Ffinding designate that apigenin suppresses the Warburg effect as well as stem-like properties in SOSP-9607 cells, which may be mediated by PI3K/Akt/mTOR signaling, therefore, providing a novel approach for osteosarcoma treatment [126].

### 3.23. Thyroid Cancer

Pioneer research found that apigenin in conjunction with TRAIL lowered cell viability and Bcl2 protein levels while increasing the proportion of dead cells when contrasted with apigenin alone. Furthermore, apigenin synergizes with TRAIL in producing cytotoxicity via the modulation of Bcl2 family proteins, and inhibition of AKT potentiates apigenin-TRAIL synergistic cytotoxicity in anaplastic thyroid carcinoma cells [95]. Apigenin anti-neoplastic properties in the papillary thyroid cancer (PTC) cell line were investigated. The results show that apigenin decreased the viability of cancer cells in a dose-dependent way. Furthermore, apigenin administration triggered autophagy in BCPAP cells, as evidenced by Beclin-1 accumulation, LC3 protein conversion, and p62 degradation. Apigenin, in particular, increased the generation of reactive oxygen species after considerable DNA damage was induced. Finally, the findings imply that molecular pathways behind apigenin-mediated autophagic cell killing exist, and that apigenin might be used as a chemotherapeutic drug [96].

### 3.24. Brain Tumour

The therapeutic effects of apigenin, quercetin, and naringenin against cancer stem cell-like phenotypes of human glioblastoma cell lines were investigated. Apigenin and quercetin significantly inhibited self-renewal capacity, like cell proliferation and clonogenicity, as well as the invasiveness of GBM stem-like cells. Apigenin inhibited the phosphorylation of c-Met and its downstream effectors, the transducer and activator of transcription 3, AKT (Protein kinase B), and mitogen-activated protein kinase, in glioblastoma stem cells. The research suggests that apigenin suppression of GBM stem cells is driven by the downregulation of the c-Met signalling pathway [97]. The influence of miR-423-5p upon the apigenin sensitivities in glioma stem cells was also explored. MiR-423-5p was highly expressed in glioma tissues and glioma stem cells. The decreased expression of miR-423-5p suppressed glioma stem cell proliferation but did not contribute to apoptosis. Furthermore, it was discovered that the combination of miR-423-5p knockdown with apigenin had a significant additive impact on reducing proliferation and increasing apoptosis in glioma stem cells. Overall, the findings suggest that downregulating miR-423-5p increases the susceptibility of glioma stem cells to apigenin via the mitochondrial route [98].

## 4. Recent Advances Displaying the Priority of Choosing Apigenin as an Anticancer Drug

Prostate cancer cells that have been co-stained for phospho-Histone H3 and DNA can be used in flow cytometric analysis to show that apigenin arrests cancer cells at the G2 phase. At the same time, apigenin lowers the mRNA and protein levels of the important regulators that control G2-M transition. The reduced transcriptional activities of the genes encoding these regulators were further confirmed by chromatin immunoprecipitation (ChIP) analysis. Understanding how apigenin inhibits the G2-M transition in cancer cells helps to explain how it works mechanistically and supports the possibility that apigenin could be used as an anti-cancer agent [127]. Inducing apoptosis through the mitochondrial pathway, apigenin increased the expression of pro-apoptotic cytochrome c, SMAC/DIABLO, and HTRA2/OMI, which aided in the activation of caspase-9 and -3. A promising method to cause cancer cell death and increase sensitivity to chemotherapy agents is to target anti-apoptotic and/or pro-apoptotic members of the apoptotic pathways [128]. The newly developed encapsulated apigenin (Ap-CH-BSA-FANPs)-treated HePG-2 cells showed the induction of apoptosis through upregulation of the p53 gene, cell cycle arrest, upregulation of caspase-9, and downregulation of the MMP9 gene and Bcl-2 protein levels. Additionally, the treatment with encapsulated apigenin increased SOD levels while lowering CAT concentrations, demonstrating the treatment’s higher antioxidant activity [129]. The purpose of a recent study is to look into how APG affects osteoblasts (hOBs) taken from a human jaw. The incubation of hOBs with growing apigenin concentrations (5, 10, 20 M) were used to measure cell viability, morphology, and proliferation over the course of 24 and 48 and 72 h. Apigenin demonstrated a stimulating effect on cell growth after demonstrating the lack of cytotoxicity and any morphological changes, with significant results using 5 M (5-APG) at 48 h. The findings demonstrated that 5-APG significantly increased the expression of the ALP and COL1 genes while speeding up osteoblastic mineralization processes [130].

## 5. Clinical Study Linked with Administration of Apigenin

In 1998, the researchers looked at how the flavonoids quercetin and apigenin affected hemostasis in healthy volunteers. It was found that apigenin at 2.5 mmol/L inhibited collagen-induced aggregation by 24–34% (NS) and ADP-induced aggregation by 22–33%. (NS) in platelet-rich plasma [131]. In a clinical study conducted by de Font-Reaulx Rojas and Dorazco-Barrag, cognitive functions in people with AD after administration containing apigenin over an extended period of time were improved [132]. The safety and efficacy of apigenin-rich chamomile oil for treating knee osteoarthritis were examined in a randomised controlled clinical trial. Patients with knee osteoarthritis who applied topical chamomile oil for three weeks saw a significant decline in their need for analgesics (acetaminophen), which improved their mobility [133].

Luteolin, apigenin, and quercetin flavonoids were found to be the most prevalent phenolic compounds in *R. coriaria* fruits. In a randomised, double-blind, placebo-controlled clinical trial, eighty hypertensive patients were given captopril (25 mg/day). The patients were split into two groups at random, and the first group was given. The first group patient was given *R. coriaria* capsules (500 mg twice a day) and captopril (25 mg once a day). The second group received placebo capsules (500 mg of starch twice daily) and 25 mg of captopril for eight weeks. The blood pressure (BP) and body mass index (BMI) of all patients were measured weekly. After eight weeks, the data showed that the *R. coriaria* group’s hypertension was significantly lower than that of the baseline and placebo groups, but that group’s BMI had not significantly changed from that of the baseline or placebo groups [134]. 

A crossover double-blind clinical trial was conducted with 100 patients. Throughout the study, each patient received two tubes of the drug and two tubes of a placebo. Patients completed visual analogue scale (VAS) questionnaires, which resulted in scores ranging from 0 to 10 (based on the intensity of the pain) over the course of 24 h. The preparation contained 0.233 mg/g of apigenin and 4.48 0.01 l/mL of chamazulene (by correcting the amount with extraction ratio). In the randomised controlled trial, 38 patients in the drug-placebo group and 34 patients in the placebo-drug group (a total of 72 patients as per protocol) finished the process (RCT). According to questionnaire results that were modified, using chamomile oleogel on the patients after 30 min caused significant (p 0.001) decreases in pain, nausea, vomiting, photophobia, and phonophobia [135].

## 6. Synergistic Effects of Apigenin in the Management of Cancer

**Figure 5:**
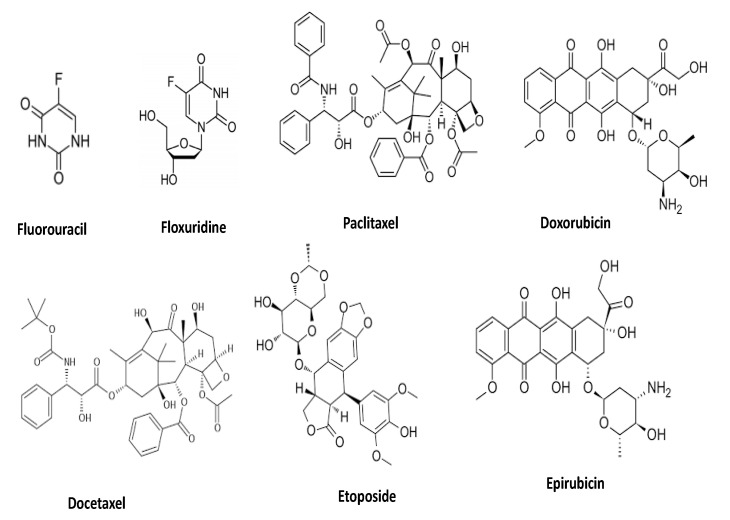
Chemical structure of clinical anti-cancer drugs.

Synergistic effects of apigenin with anti-cancer drugs (Figure 5) has been confirmed based on experimentation (Table 3). HeLa cells were administered with multiple dosages of apigenin, paclitaxel, or both, and cell viability of HeLa cells was evaluated. The study found that both apigenin and paclitaxel caused dose-dependent cytotoxicity, with apigenin producing a 29% drop in cell viability and paclitaxel inducing a 24% reduction. The combination index at the dosage of 15 µM apigenin and 4 nM paclitaxel was 0.3918 ± 0.0436, showing the synergistic effects of apigenin and paclitaxel. It was further reported that administration of a combination of apigenin and paclitaxel to cancer cells promoted the cytotoxicity dramatically. The unique combination of apigenin and paclitaxel produced comparable outcomes in cancer cells including A549 and Hep3B cells [136]. The consequences of 5-fluorouracil and apigenin combination therapy for proliferation and apoptosis in human breast cancer cells were studied. The MDA-MB-453 cells, which have been shown to overexpress ErbB2, were resistant to 5-fluorouracil; 5-fluorouracil exhibited a small dose-dependent anti-proliferative effect. Surprisingly, a combination therapy with apigenin significantly reduced resistance. Furthermore, cellular proliferation was greatly decreased by 5-fluorouracil with apigenin in comparison with 5-fluorouracil alone. It was further reported that 5-fluorouracil alone had few effects on ErbB2 levels, but 5-fluorouracil with apigenin had a considerable effect. Finally, the results indicate that 5-fluorouracil has significant synergistic effects with apigenin to suppress cell proliferation, while it also causes apoptosis via the down-regulation of Akt signalling and ErbB2 expression [61]. 

Apigenin was tested for its ability to increase the chemosensitivity of hepatocellular carcinoma cells as well as the hepatocellular carcinoma xenograft model in responding to 5-fluorouracil (5-FU). Apigenin at sub-toxic doses was shown to substantially increase the cytotoxicity of 5-fluorouracil in hepatocellular cancer cells. In vivo, apigenin and 5-fluorouracil combination therapy effectively suppressed the development of xenograft hepatocellular carcinoma tumours. Moreover, the administration of hepatocellular carcinoma cells with a combination of apigenin and 5-fluorouracil raised the concentration of reactive oxygen species, which was followed by a drop in mitochondrial membrane potential. Further, the researchers discovered that the cytotoxicity of 5-fluorouracil in hepatocellular carcinoma becomes enhanced by apigenin because of its capability to inhibit ROS-mediated drug resistance as well as the concurrent promotion of mitochondrial apoptotic pathways [137]. 

Another study result demonstrated that Apigenin inhibits the growth of head and neck cancer cells and induces cell cycle arrest in the G2/M phase. It has been reported that BNLCL2 cells are protected by apigenin for oxidative damage, and it is also able to prevent cancer. In addition, the concentration of intracellular reactive oxygen species becomes raised by apigenin. However, it causes lowering of the levels of glutathione, as well as the stimulation of cell apoptosis via tumor necrosis factor receptors. In addition, combining apigenin with 5-fluorouracil or cisplatin promotes SCC25 cells to die dramatically. Therefore, the researchers reported that apigenin causes cancer cell death through the up-regulating of both TNF-R and TRAIL-R signalling pathways, and it further exerts a cumulative impact on cell growth inhibition when combined with 5-Fu or cisplatin [138]. Apigenin and curcumin synergistically induced cell death and apoptosis and blocked cell cycle progression at G_2_/M phase of A549 cells [139]. 

## 7. Bioavailability of Apigenin

Apigenin and other medicinal plants have been reported to have a significant effect on cancer and disease prevention as well as its progression [144,145]. The use of natural products derived from plants are recommended to be included into any existing preventative and treatment approaches for the management of diseases [146]. Various phytochemicals such as flavonoids or active compounds are reported to be responsible for the therapeutic effects of medicinal plants [147,148].

In other words, bioavailability is a measure of the rate and portion of the initial dose of a drug that effectively reaches either the site of action or the bodily fluid domain from which the drug’s intended targets have unhindered access [149,150,151]. Hydrophobic compounds mostly show poor bioavailability when given to animals due to their low absorption capacity, and this results in low amounts of the drug getting to the target tumor as well as the increased toxicity to normal tissues [152]. From a nutritional viewpoint it can also be well-defined as the amount of absorption, digestion, metabolism, and excretion of a compound after the ingestion of food [153]. Normally, when drugs are consumed orally, dietary polyphenols are primarily absorbed and metabolized in the small intestine [154,155,156], and only a small proportion of it is absorbed in the stomach [157]. Another finding has demonstrated that the solubility of apigenin in aqueous solutions, vary in the range of 0.001 to 1.63 milligrams per milliliter in nonpolar solvents [158], whereas, its solubility in a phosphate buffer has been found to be 2.16 µg/mL [159]. Apigenin oral bioavailability is comparably limited owing to its poor aqueous solubility (2.16 g/mL in water) [160], which has significantly hampered its future research clinical development. Apigenin has a half-life of 91.8 h in blood, a distribution volume of 259 mL, and a plasmatic clearance of 1.95 mL/h, indicating that it is absorbed and eliminated slowly in the body [161].

The limited solubility of AP prevents its clinical use. Various components of the drug delivery system such as liposomes, an apigenin-loaded self-microemulsifying drug delivery system, apigenin-loaded polymeric micelle, carbon nanopowder, a nanosuspension, and a nanocrystal made using a supercritical antisolvent process have been suggested by researchers. This drug delivery system has been shown to help increase the bioavailability of apigenin. Furthermore, solid dispersion has been frequently used to improve the solubility and dissolution of drugs that are poorly water-soluble as well as to address the issues with stability and dosing. The solubility, dissolution, and oral bioavailability of apigenin are all improved by a novel mesoporous silica nanoparticle drug carrier [162].

## 8. Cytotoxicity of Apigenin

With little to no toxicity to normal cells, apigenin exhibits significant cell cytotoxicity, specifically against various types of cancer cells. It has also been demonstrated that these selective anti-cancer effects suppress cancer stem cells (CSCs) in a variety of cancers [81]. The type of cancer cell determines how autophagy functions in apigenin-induced cytotoxicity. According to most reports, apigenin-triggered autophagy mediates the cancer cells’ acquired resistance to cell apoptosis. This is demonstrated by the increased cell apoptosis induced by apigenin when used in combination with autophagy inhibitors. In this situation, autophagy mitigates the cytotoxicity that apigenin-induced cancer cells experience. In contrast, human papillary thyroid carcinoma BCPAP cells undergo autophagic cell death due to autophagy [96]. Utilizing a 3-MA autophagy inhibitor substantially increased apigenin-induced apoptosis, which indicates that the tumor-protective effects of apigenin’s autophagy are also still observable [163]. Autophagy’s role in apigenin-induced cytotoxicity is determined by cancer cell type. As shown by increased cell apoptosis caused by apigenin when combined with autophagy inhibitors, most reports suggest that apigenin-triggered autophagy mediates the cancer cells’ acquired resistance to cell apoptosis. In this case, autophagy protects cancer cells from apigenin-induced cytotoxicity [164]. 

## 9. Advantage of Apigenin over FDA Approved Anticancer Drugs

The side effects of oncological medications are notoriously bad, and they struggle with issues like low response rates and drug resistance. Anticancer drugs frequently have a variety of unfavorable side effects that negatively affect cancer patients’ organs and ultimately make their symptoms worse. Understanding these toxicities and negative effects is crucial. Unprecedented difficulties are presented by rising drug prices and the decreasing number of truly effective medications that the US Food and Drug Administration (FDA) has approved. The negative effects and acquired drug resistance of synthetic small molecule compounds have raised more and more questions as their use and understanding have grown. Therefore, natural and edible small molecules such as flavones, which are thought to have remarkable physiological effects, low toxicity and non-mutagenic properties in the human body, have gained more and more interest in anti-cancer agent development. Apigenin appears to have the potential to be developed either as a dietary supplement or as an adjuvant chemotherapeutic agent for cancer therapy. Apigenin has been shown various human cancers in vitro and in vivo through multiple biological mechanisms, including inducing cell cycle arrest, triggering cell apoptosis and autophagy, stimulating an immune response, and suppressing cell migration and invasion [164,165].

## 10. The Binding Modes of Apigenin 

The use of computational methods is a useful strategy for dealing with chemical issues. For example, protein-ligand binding can be simulated using molecular docking. Docking produces significant results used for binding-mode predictions despite its shortcomings and simplifications. Regarding the explanation of ligand binding and protein behaviour, molecular dynamics is receiving more and more attention. A study was conducted to evaluate the interpretation of some forms of apigenin with the target MAPK P38 (P38 Mitogen activated protein kinase) using docking program GEMDOCK. Apigenin showed close orientation towards MAPK P3 with binding energy −100 (kcal/mol), and H bond interacting residues including SER 251, ASP292, ASP 294 [166]. In another study, the binding mode between apigenin and STAT3 was examined. Apigenin and WP1099 had binding energies to STAT3 of −8.9 and −7.4 kcal/mol, respectively. The backbone of Arg247 in STAT3 subunit A and apigenin were found to form a hydrogen bond, with a donor-acceptor distance of 3.0 (Su et al., 2020). In an in-silico study, apigenin was found to bind to the NBDs of P-glycoprotein and ABCB5. Hence, apigenin may compete with ATP for NBD-binding, leading to energy depletion to fuel the transport of ABC transporter substrates [167].

To ascertain the efficiency of apigenin and PS1145, an IKK inhibitor, in reducing kinase activity, a study was conducted in silico docking studies. According to the docking results, both apigenin and PS1145 were docked to the deep cleft in the IKKα structure. Apigenin’s docked conformation shows two fused aromatic rings at the base of the pocket and one aromatic ring sticking outward. Two hydrogen bonds hold apigenin in place inside the pocket. One of the H bonds connects the side chain of Asp165 to one of the hydroxyl groups in the buried phenyl ring. However, the other H bond connects the carbonyl oxygen of apigenin to the backbone of Cys98 [93]. By using molecular docking calculations against the acetylcholinesterase (AChE), butyrylcholinesterase (BChE), amyloid precursor protein (APP), and 42-residue beta-amyloid peptide (A), it was determined whether apigenin-7-glucoside (A7G) and luteolin-7-glucoside (L7G) could be used as multi-targeted agents in AD. A7G and L7G exhibited very high binding affinity (−9.42 and −9.60 kcal/mol for A7G; −9.30 and −9.90 kcal/mol for L7G) to AChE and BChE, respectively, while the affinities of these two flavonoid glycosides towards APP and Aβ peptide (−6.10 and −6.0 kcal/mol for A7G; −6.30 and −6.10 kcal/mol for L7G) were moderately strong [168].

A study used a constant DNA concentration of 6.25 mM and various drug/DNA (phosphate) ratios of 1/40 to 1 to examine the interactions of morin (Mor), naringin (Nar), and apigenin (Api) with calf thymus DNA in aqueous solution under physiological conditions. The ligand binding modes, the binding constant, and the stability of DNA in flavonoid-DNA complexes in aqueous solution were identified using FTIR and UV-Vis spectroscopic techniques. According to spectroscopic evidence, flavonoids bind to the DNA duplex both internally and externally, with overall binding constants of Kmorin = 5.99 × 103 M^−1^, Kapigenin = 7.10 × 104 M^−1^, and Knaringin = 3.10 × 103 M^−1^ [169].

In a study, quercetin and apigenin were found to inhibit the formation of β-hematin. Molecular docking and virtual screening were performed to understand the mechanism of ligand binding and to identify potent calcium transporter inhibitors. All of the selected compounds in the *A. digitata* had binding energy ranging between −6.5 kcal/mol and −7.1 kcal/mol. Among the two chemical constituents, apigenin has the highest docking score along with the highest number of hydrogen bonds formed when compared to quercetin [170]. 

## 11. Conclusions

Cancer is the most commonly diagnosed type of disease and a major cause of death worldwide. The current mode of treatment causes adverse effects such as appetite loss, constipation, bleeding, diarrhea, edema, fatigue, hair loss, and kills normal cells. Apigenin acts as a dynamic source of drugs due to its having lesser side effects, and it also appears to have a role in the reduction of resistance against cancer therapy. The antioxidant potentiality of apigenin shows that it plays a vital role in the inhibition of free radical formation, the modulation of oxidative stress, inflammation, and finally in managing cancer’s development and progression. This flavonoid seems to have anti-cancer activities via the modulation of a various cell signaling pathways including angiogenesis, apoptosis, cell cycle and various other genetic pathways. Further studies based on clinical trials are needed to explore the role of apigenin in cancer management and to explain the possible potential mechanisms of action in this area.

## Figures and Tables

**Table 1:** Major mechanism of apigenin in cancer management through modulating gene activity.

Genes	Mechanism	Refs.
TNFα	Apigenin downregulates the TNFα mediated release of chemokines and suppresses Interlukin-6 and interlukin-1α.	[16]
NF-κB	Apigenin can regulate the production as well as the gene expression of mucin via regulating the nuclear factor-κB signaling pathway in airway epithelial cells.	[17]
P53	The higher detection of Bax was related to greater p53 accumulation. It suggested that apigenin boosted the cytotoxic impact of cisplatin by inducing p53 accumulation and p53-regulated proapoptotic gene expression.	[18]
Bcl-2 and Bax	Apigenin was shown to be associated with a decrease in Bcl-xL and Bcl-2 levels, as well as an increase in the active form of the Bax protein.	[19]
Bax/Bcl-2	PARP cleavage DNA fragmentation revealed that apoptosis was induced by apigenin treatment. These effects were discovered to be linked to a rise in the Bax/Bcl-2 ratio, which indicates a change favoring apoptosis.	[20]
VEGF	Apigenin inhibited HIF-1alpha and vascular endothelial growth factor expression in the tumor tissues, showing an inhibitory effect of apigenin on angiogenesis.	[21]
VEGF	Apigenin demonstrated that it had a role in the inhibition of the hypoxia-induced expression of vascular endothelial growth factor mRNA	[22]
P53	Apigenin exposure induces G2/M phase cell cycle arrest, DNA damage, apoptosis and p53 accumulation, which collectively suppressing cancer cell proliferation in vitro and in vivo.	[23]
Cyclin B, cyclin A, and cyclin-dependent kinase-1	Apigenin inhibited the expression of cyclin B, cyclin A, and cyclin-dependent kinase-1, all of which are involved in the cell cycle G2-to-M transition.	[24]
PI3K/Akt/mTOR pathway	Apigenin plays a role in the induction of apoptosis as well as autophagy via the inhibition of the pathway of PI3K/Akt/mTOR	[25]
PI3K/Akt/FoxO-signaling pathway	Apigenin inhibited prostate tumorigenesis in transgenic prostate adenocarcinoma via the FoxO/PI3K/Akt signaling cascade.	[26]
IKKα	Apigenin straight binds with IKKα, decreases IKKα kinase activity, and subdues NF-ĸB/p65 activation in cancer cells much more effectively than than an IKK inhibitor	[27]
ERK	TRAIL-induced antitumor activity in lung cancer cells by the treatment of apigenin through inhibition of the ERK, Nuclear factor F-κB, and AKT prosurvival regulators	[28]
ERK	Apigenin suppressed AKT and ERK activation. Moreover, it enhanced ABT-263-induced antitumor activity in colon cancer cells via apigenin through the inhibition of the AKT, Mcl-1 as well as ERK prosurvival regulators	[29]
ERK	Phosphorylation of AKT, P70RSK, and S6 was decreased by apigenin while the phosphorylation of ERK1/2 and P90RSK was increased by apigenin treatment	[30]
STAT3	Apigenin activated p53 that induced catalase, a reactive oxygen species scavenger enzyme, and inhibited *Signal transducer and activator of transcription 3*, the most important pro-survival pathway in primary effusion lymphoma.	[31]
STAT3	apigenin efficiently repressed *Signal transducer and activator of transcription 3*phosphorylation, decreased STAT3 nuclear localization and repressed *Signal transducer and activator of transcription 3*transcriptional activity	[32]
MMP	Apigenin down-regulated *Signal transducer and activator of transcription 3*target genes MMP-2, MMP-9 and vascular endothelial growth factor that participate in cell migration and invasion	[32]
EFGR	Apigenin and Cetuximab could decrease the expressions of p- epidermal growth factor receptor, p-Akt, p-*Signal transducer and activator of transcription 3* and Cyclin D1	[33]
Wnt/β-catenin signaling pathway	Apigenin significantly suppressed colorectal cancer cell proliferation, migration, invasion and organoid growth through inhibiting the Wnt/β-catenin signaling pathway	[34]

**Table 2:** Apigenin plays a significant role in various types of cancer inhibition.

Types of Cancer	Outcome of the Study	Refs.
Breast cancer	Apigenin exhibited potent growth-inhibitory activity in breast cancer cells. Moreover, the induction of apoptosis was also seen in neu/HER2 overexpressing breast cancer cells	[59]
Breast cancer	Treatment with apigenin significantly inhibited the proliferation of human breast cancer cells in a dose- and time-dependent fashion	[60]
Breast cancer	5-fluorouracil acts synergistically with apigenin, inhibiting cell growth as well as causing the induction of apoptosis through the down-regulation of Akt signaling and ErbB2 expression	[61]
Cervix cancer	Apigenin inhibited the growth of cancer cells through the apoptotic pathway. Also, apigenin significantly induced apoptosis and decreased the viability of cancer cells	[62]
Cervix cancer	Apigenin exerts its anti-tumorigenic effect in vivo through the inhibition of tumour cell infiltration of the healthy tissue	[63]
Ovarian cancer	Apigenin suppressed the expression of the inhibitor of differentiation or DNA binding protein 1 via activating transcription factor	[64]
Ovarian cancer	Caspase-9 activity was significantly increased in apigenin-treated cancer cells and the cell cycle at the G_2_/M phase was arrested by apigenin	[65]
Ovarian cancer	Apigenin inhibited the self-renewal capacity of SKOV3-derived SFCs and was involved in downregulating the expression of Gli1 by the preventing of CK2α	[66]
Pancreas cancer	Apigenin exerted a stronger cytotoxic effect against cancer cells. Moreover, the higher cytotoxicity of apigenin correlated with a reduction of mutant p53 expression and the induction of higher levels of intracellular reactive oxygen species	[67]
Pancreas cancer	Apigenin reduced levels of cyclin A, cyclin B, phosphorylated forms of cdc2 and cdc25 and induced G2/M phase cell cycle arrest	[68]
Pancrease cancer	Apigenin induced apoptosis and reduced cell growth	[69]
Bile duct cancer	Apigenin demonstrates an induction of apoptosis and growth inhibition in cancer cells	[70]
Colon cancer	Apigenin inhibits the m-TOR/PI3K/AKT signalling pathway in cisplatin-resistant cancer cells	[71]
Colon cancer	Apigenin inhibits the epithelial-mesenchymal transition, invasion and migration of cancer cells based on in vitro and in vivo	[72]
Colon cancer	Apigenin induces the apoptosis of cancer cells by preventing the phosphorylation of signal transducer and activator of transcription 3 and therefore downregulates the anti-apoptotic proteins Bcl-xL and Mcl-1	[73]
Urinary bladder cancer	Apigenin inhibited the migration, invasion and decreased proliferation of bladder cancer cells in a dose- and time-dependent way that was linked with the induction of apoptosis and G2/M phase cell cycle arrest	[74]
Urinary bladder cancer	Apigenin can cause anti-invasion effects via inhibiting uPAR expression	[75]
Urinary bladder cancer	Apigenin inhibited actin polymerization, which emphasizes muscle contraction and cell migration.	[76]
Liver cancer	Apigenin induced autophagy and apoptosis through the inhibition of the mTOR/PI3K/Akt pathway	[25]
Liver cancer	Apigenin induced G1 arrest in a cancer in a dose-dependent fashion	[77]
Liver cancer	Apigenin meaningfully enhanced the sensitivity of doxorubicin, induced expression of miR-520b and inhibited ATG7-dependent autophagy	[78]
Renal cancer	Apigenin treatment reduced tumor volume and growth in vivo. Finally, apigenin exposure induces DNA damage, p53 accumulation and apoptosis and G2/M phase cell cycle arrest	[23]
Esophagus cancer	Apigenin meaningfully and dose-dependently inhibited cell proliferation and promoted apoptosis and suppressed vascular endothelial growth factor expression and tumor-initiated angiogenesis	[79]
Esophagus cancer	Membrane toxicity, including enhanced membrane permeability, membrane ultrastructure damages, showed important roles in apigenin induced cancer cell apoptosis	[80]
Head and neck cancer	Apigenin significantly decreased cancer cell viability in a dose- and time-dependent fashion	[81]
Head and neck cancer	Apigenin as well as kaempferol treatment decreased the viability of cells in vitro. In vivo apigenin treatment significantly increased the tumor size	[82]
Lung cancer	Apigenin sensitized lung cancer cells to TRAIL-induced apoptosis through upregulating the levels of death receptor 4 and death receptor 5	[28]
Oral cancer	The anticancer potential of apigenin in an oral squamous cell carcinoma was noted, proposing that it may be a very hopeful chemopreventive agent	[83]
Lymphoma	Apigenin leads to an important reduction of the expression of pro-proliferative pathways in mTOR/PI3K to inhibit cancer cell survival	[84]
Lymphoma	Apigenin activated p53 that induced catalase, a reactive oxygen species scavenger enzyme, and inhibited the signal transducer and activator of transcription 3	[52]
Skin cancer	Apigenin inhibited cell migration, invasion and induced G2/M phase arrest and apoptosis	[85]
Mesothelioma	API-induced apoptosis was continued by the increase of Bax/Bcl-2 ratio, activation of both caspase 9 and caspase 8 and the increase of p53 expression,	[86]
Adenoid cystic carcinoma-	Apigenin inhibits carcinoma-2 cell growth in a dose- and time-dependent way. Treatment with apigenin also induced apoptosis and G_2_/M-phase arrest in a dose- and time-dependent fashion	[87]
Myeloma	Apigenin treatment downregulated the expression of the antiapoptotic proteins and survivin that finally induced apoptosis in multiple myeloma cells	[88]
Leukemia	Apigenin is a potential chemo-preventive agent due to the induction of leukemia cell-cycle arrest	[89]
Leukemia	Administration of apigenin resulted in attenuation of tumor growth in xenografts U937 convoyed inactivation of Akt as well as activation of JNK	[90]
Prostate cancer	Apigenin suppressed the proliferation as well as inhibited the migration and invasive in a dose- and time-dependent way	[91]
Prostate cancer	Apigenin treatment caused the significant decrease in cell viability and the induction of apoptosis	[92]
Prostate cancer	Apigenin caused cell cycle arrest	[93]
Osteosarcoma	Apigenin meaningfully decreased cell viability and efficiently induced apoptosis via the activations of caspase and BAX	[94]
Osteosarcoma	Apigenin inhibits the tumor growth of osteosarcoma cells via inactivating Wnt/β-catenin signaling	[55]
Thyroid cancer	Apigenin synergizes with TRAIL via the regulation of Bcl2 family proteins	[95]
Thyroid cancer	Apigenin enhanced production of reactive oxygen species following the induction of significant damage of DNA	[96]
Brain cancer	The *glioblastoma stem* cell inhibition effect of apigenin may be caused via the downregulation of the c-Met signaling pathway	[97]
Brain cancer	Downregulation of miR-423-5p enhances the sensitivity of glioma stem cells to apigenin through the mitochondrial pathway	[98]

**Table 3:** Synergistic effect of apigenin with anti-cancer drugs.

Types of Tumours	Anti-Cancer Drugs	Outcome of the Study	Refs.
Cervix cancer	Paclitaxel	Both apigenin and paclitaxel dose dependently induced cytotoxicity	[136]
Cervix cancer	IFN gamma	Apigenin increased the anticancer activity of IFNgamma in cancer cell lines through targeting cylin-dependent kinase 1.	[18]
Breast cancer	5-fluorouracil	Combined treatment with apigenin meaningfully decreased the resistance. Cellular proliferation was significantly inhibited in cells exposed to 5-fluorouracil and apigenin	[61]
Breast cancer	Cisplatin	Apigenin treatment alone caused a 10–30% low viability of cells whereas cisplatin alone caused a 3%viability at a low dose. The co-treatment of apigenin and cisplatin increased the inhibitory effects of cisplatin	[140]
Liver cancer	5-fluorouracil	In vivo, the combined treatment with apigenin and 5-fluorouracil significantly inhibited the growth of liver cancer xenograft tumours. Moreover, the treatment of liver cancer cells with apigenin as well as 5-fluorouracil increased levels of reactive oxygen species	[79]
Head and neck cancer	5-fluorouracil	The ombination of apigenin with 5-fluorouracil or cisplatin induces the dramatic death of cancer cells.	[137]
Ovarian cancer	Taxol	Apigenin, when used with cisplatin, inhibited cell proliferation and promoted mitogen-activated protein kinase activation and successive phosphorylation of p53	[140]
Laryngeal carcinoma	Cisplatin	Apigenin may significantly reduce the levels of GLUT-1, GLUT-1 mRNA, and p-Akt proteins in cisplatin-treated cancer cells	[141]
Pancreatic cancer	5-fluorouracil	The pretreatment of pancreatic cancer cells with low concentrations of Api or Lut effectively aid in the anti-proliferative activity of chemotherapeutic drugs	[142]
Leukemia	Doxorubicin and etoposide	Doxorubicin and etoposide in combination with polyphenols synergistically reduced ATP levels, increased S and/or G2/M phase cell cycle arrest and induced apoptosis	[143]
Colon cancer	ABT-263	A novel strategy to enhance ABT-263-induced antitumor activity in cancer cells by apigenin via the inhibition of the Mcl-1, AKT, and ERK prosurvival regulators.	[29]

## Data Availability

The data used to support the findings of this study are included within the article.
